# Supplementary figures and images for: Neural circuitry of a polycystin-mediated hydrodynamic startle response for predator avoidance
Source: eLife. 2018 Dec 14;7:e36262. doi: 10.7554/eLife.36262 (PMC6294549; doi:10.7554/eLife.36262)

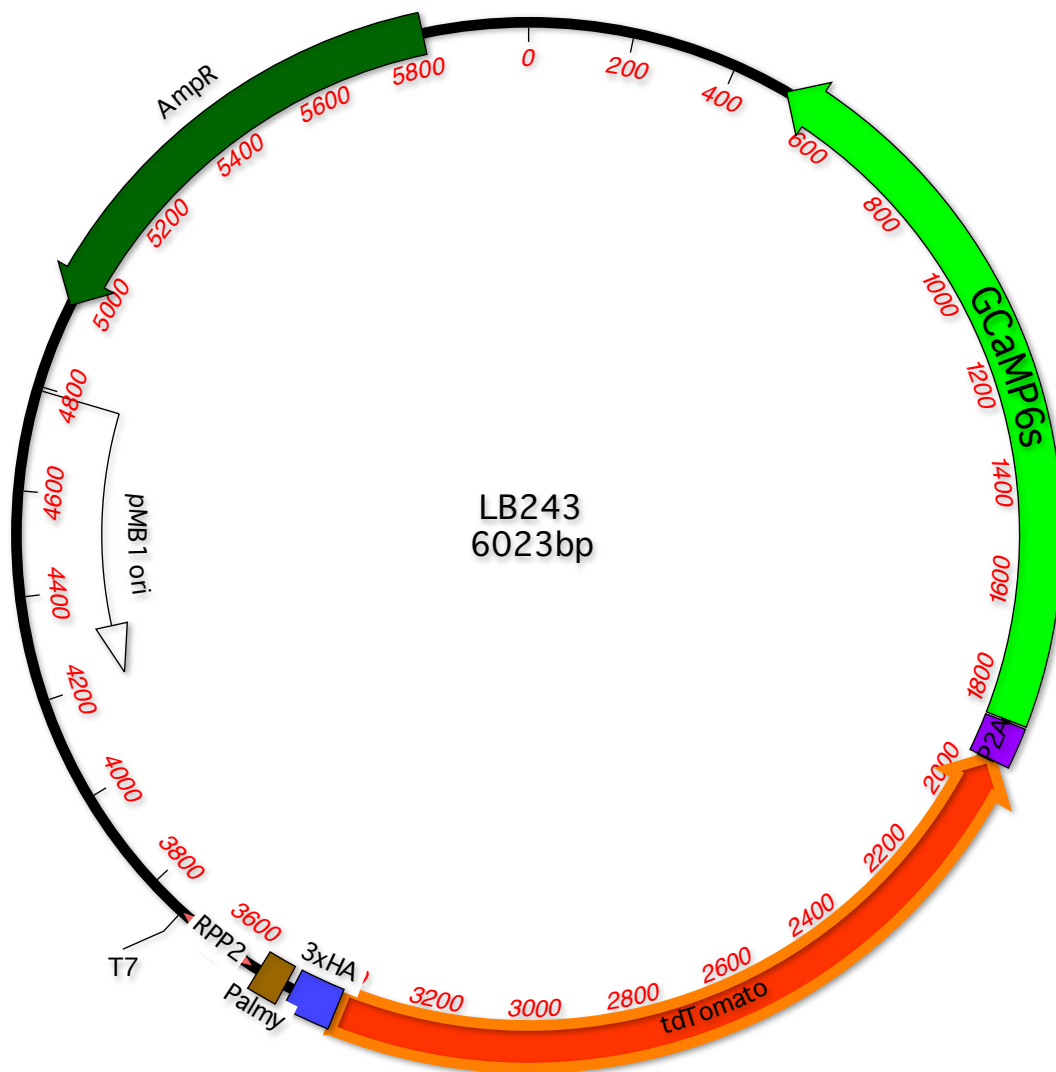

Supplement: Figure 2—source data 2. [file elife-36262-fig2-data2.zip › LB243_Palmi-tdtomato-P2A-GCaMP6s.pdf]
