# Supplementary material for: Neural circuitry of a polycystin-mediated hydrodynamic startle response for predator avoidance
Source: eLife. 2018 Dec 14;7:e36262. doi: 10.7554/eLife.36262 (PMC6294549; doi:10.7554/eLife.36262)
Supplement: Transparent reporting form [file elife-36262-transrepform.docx]

***eLife’s* transparent reporting form**

We encourage authors to provide detailed information *within their submission* to facilitate the interpretation and replication of experiments. Authors can upload supporting documentation to indicate the use of appropriate reporting guidelines for health-related research (see [EQUATOR Network](http://www.equator-network.org/%20)), life science research (see the [BioSharing Information Resource](https://biosharing.org/)), or the [ARRIVE guidelines](http://www.plosbiology.org/article/info:doi/10.1371/journal.pbio.1000412) for reporting work involving animal research. Where applicable, authors should refer to any relevant reporting standards documents in this form.

If you have any questions, please consult our Journal Policies and/or contact us: [editorial@elifesciences.org](mailto:editorial@elifesciences.org).

**Sample-size estimation**

- You should state whether an appropriate sample size was computed when the study was being designed
- You should state the statistical method of sample size computation and any required assumptions
- If no explicit power analysis was used, you should describe how you decided what sample (replicate) size (number) to use

Please outline where this information can be found within the submission (e.g., sections or figure legends), or explain why this information doesn’t apply to your submission:

Sample size was not explicitly determined prior to our experiments. Instead single larva experiments (Figure 1,2E-G, and 4G,H) were designed to include data from 3 or more specimens, each preferably from different crossings and performed in different days.

Batch experiments (Figure 4E,F and Figure 1-figure supplement 1A,B) were designed to include as many batches as time limitations allowed, but testing no less than 3 batches, and spreading the experiments across different days.

Experiments using qualitative techniques such as whole-mount *in situ* hybridization, immunofluorescence and scanning electron microscopy imaging (Figure 2A-C,D, Figure 3, Figure 3 -figure supplement 2,3) were designed to produce internally consistent data to allow the assessment of general patterns.

Connectome data: due to the significant time and resources needed to generate these data we only analyzed one specimen.

**Replicates**

- You should report how often each experiment was performed
- You should include a definition of biological versus technical replication
- The data obtained should be provided and sufficient information should be provided to indicate the number of independent biological and/or technical replicates
- If you encountered any outliers, you should describe how these were handled
- Criteria for exclusion/inclusion of data should be clearly stated
- High-throughput sequence data should be uploaded before submission, with a private link for reviewers provided (these are available from both GEO and ArrayExpress)

Please outline where this information can be found within the submission (e.g., sections or figure legends), or explain why this information doesn’t apply to your submission:

Startle Kinematics: We defined as biological replicate each stimulation level and larva tested. We minimized the dependence between stimulation trials by randomizing stimulus strength and allowing ample recovery time. This is indicated in the corresponding Materials & Methods section.

In the calcium imaging experiments biological replicate were defined as in the startle kinematics experiments (see above).

Touch phenotyping: Each individually tested larva was counted as a biological replicate.

Predator assay: Each prey-predator incubation experiment is treated as a biological replicate.

Sample size for each experiment is indicated in the relevant figure legends.

All data generated from completed experiments were included in the analysis. Outliers are included in all tables and plots in the article.

**Statistical reporting**

- Statistical analysis methods should be described and justified
- Raw data should be presented in figures whenever informative to do so (typically when N per group is less than 10)
- For each experiment, you should identify the statistical tests used, exact values of N, definitions of center, methods of multiple test correction, and dispersion and precision measures (e.g., mean, median, SD, SEM, confidence intervals; and, for the major substantive results, a measure of effect size (e.g., Pearson's r, Cohen's d)
- Report exact p-values wherever possible alongside the summary statistics and 95% confidence intervals. These should be reported for all key questions and not only when the p-value is less than 0.05.

Please outline where this information can be found within the submission (e.g., sections or figure legends), or explain why this information doesn’t apply to your submission:

The statistical test performed in the predator-prey experiment (Fig. 5) is described in the main text (section “*PKD1-1* and *PKD2-1* are required for the startle response”) and the P-value obtained is reported in the figure legend.

Statistical tools used to assess phylogenetic branch support are reported in the relevant methods section, and the cutoff value to define resolved dichotomies stated in the figure legend.

For each experiment, the measure of center is indicated graphically (in line plots for Calcium imaging, or in boxplots for kinematics experiments) and in the corresponding figure legends.

(For large datasets, or papers with a very large number of statistical tests, you may upload a single table file with tests, Ns, etc., with reference to sections in the manuscript.)

**Group allocation**

- Indicate how samples were allocated into experimental groups (in the case of clinical studies, please specify allocation to treatment method); if randomization was used, please also state if restricted randomization was applied
- Indicate if masking was used during group allocation, data collection and/or data analysis

Please outline where this information can be found within the submission (e.g., sections or figure legends), or explain why this information doesn’t apply to your submission:

We randomized stimulus intensity during the kinematics experiments as described in the Materials and Methods section.

We assigned phenotypes blindly (i.e. prior to knowing genotype) during the experiments shown in Figure 4.

**Additional data files (“source data”)**

- We encourage you to upload relevant additional data files, such as numerical data that are represented as a graph in a figure, or as a summary table
- Where provided, these should be in the most useful format, and they can be uploaded as “Source data” files linked to a main figure or table
- Include model definition files including the full list of parameters used
- Include code used for data analysis (e.g., R, MatLab)
- Avoid stating that data files are “available upon request”

Please indicate the figures or tables for which source data files have been provided:

Source data are provided for 6 main figures in table format.

Sequence data and sequence alignments are also included (Figure 3 figure supplement 1 source data 1).

Code used for data analysis is available at: <https://github.com/JekelyLab/Bezares_et_al_2018>.
